# Hypertrophy of the Maxilla

**Published:** 1872-05

**Authors:** 


### EDITORIAL DEPARTMENT.

*Hypertrophy of the Maxilla.*—Dr. Geo. S. Staples, of Corinth, Miss., sends us the following case:

"I will relate to you a case in practice, and would like to know if you could divine any cause for the unnatural growth in the case. Mrs. B — aged about thirty, had all her teeth removed for the purpose of having a full set inserted. She applied to some itinerant—a class of which we are blessed with in this portion of country—to insert the teeth, when he informed her 'that owing to two large *knots* on the upper gums it would be impossible to fit a plate to her mouth.'

"She afterwards applied to several others of the same class with the same results, until she had almost despaired of getting teeth at all. Happening to be in the neighborhood she sent for me to examine her mouth. I did so and found it to be a case of exostosis of the alveolar process, in two different places; one just over the left canine tooth, and the other extending over the second and third molars on the right side, each as large as the end of a man's thumb, and the latter an inch in length. They had formed there after the extraction of her teeth and continued to grow all the time. Having nothing better at hand I removed the protuberances with a gum lancet and a pair of fang forceps. Six weeks afterwards I inserted a full set of teeth which she is still wearing (it being four years since) with perfect satisfaction, with no sign of the return of the disease. Her teeth I suppose were very much decayed, with a large accumulation of tartar, when extracted."

[Hypertrophy of the Maxilla or Hyperostosis, consists of some inflammatory affection of the periosteum, which causes the deposit of new bone. Anything capable of irritating the investing membrane of the bone may be regarded as a cause; and in the case given above by Dr. Staples, the condition of the teeth previous to their removal was no doubt the primary cause of the hyperostosis.—Ed.]

